# Centrosome dysfunction: a link between senescence and tumor immunity

**DOI:** 10.1038/s41392-020-00214-7

**Published:** 2020-06-30

**Authors:** Qi Wu, Bei Li, Le Liu, Shengrong Sun, Si Sun

**Affiliations:** 1 Department of Breast and Thyroid Surgery, Renmin Hospital of Wuhan University, Wuhan, Hubei China; 2 Department of Pathology, Renmin Hospital of Wuhan University, Wuhan, Hubei China; 3 Center of Ultramicroscopic Pathology, Renmin Hospital of Wuhan University, Wuhan, Hubei China; 4 Department of Clinical Laboratory, Renmin Hospital of Wuhan University, Wuhan, Hubei China

**Keywords:** Senescence, Tumour immunology, Senescence

## Abstract

Centrosome aberrations are hallmarks of human cancers and contribute to the senescence process. Structural and numerical centrosome abnormalities trigger mitotic errors, cellular senescence, cell death, genomic instability and/or aneuploidy, resulting in human disorders such as aging and cancer and affecting immunity. Interestingly, centrosome dysfunction promotes the secretion of multiple inflammatory factors that act as pivotal drivers of senescence and tumor immune escape. In this review, we summarize the forms of centrosome dysfunction and further discuss recent advances indicating that centrosome defects contribute to acceleration of senescence progression and promotion of tumor cell immune evasion in different ways.

## Introduction

In animal cells, the centrosome is the major microtubule organizing center (MTOC). Centrosomes promote the production of bipolar mitotic spindles and supply a matrix of primary cilia in various cell types. In addition to these structural functions, centrosomes and primary cilia have also evolved into essential signaling hubs.^1^ In normal cells, a pair of centrioles comprises the centrosome, which is embedded in the pericentriolar material (PCM), an electron-dense amorphous matrix. The PCM supplies sites for microtubule nucleation, thus determining the number and composition of microtubules during the cell cycle. Large-scale proteomic studies have revealed that there are more than 200 centrosome-associated proteins;^2^ however, the exact functions of most of these proteins are still unknown and require further investigation.

The structure, function and number of centrosomes are strictly controlled within cells. To form an effective bipolar mitotic spindle, the centrosome must duplicate in the S phase, and further centrosome maturation occurs when additional PCM material is recruited during the G2 phase of the cell cycle. Finally, the two centrosomes must separate upon mitotic entry. Like DNA replication, centrosome duplication is semiconservative and occurs only once per cell cycle. A rigorous regulatory network ensures that the two processes are carried out in an ordered manner during the late G1 phase.^2^ Therefore, centrosome number determination and centrosome duplication are associated in each cell. Proper centrosome duplication, maturation and separation are fundamental for the formation of bipolar spindles and, therefore, for faithful chromosome segregation. As a consequence, the accurate inheritance of genetic material might be hindered by centrosome dysfunction (CD).^3^ Furthermore, centrosome abnormalities can lead to chromosomal instability (CIN), an ongoing process of gain and/or loss of whole chromosomes.^4,5^ In turn, this can lead to aneuploidy, a state in which too many or too few chromosomes are present.^6^ Therefore, abnormal chromosome content, a biomarker of human cancers, is associated with aneuploidy and CIN.^4,7^

Initially, cellular senescence was identified as a mostly irreversible type of growth arrest that occurs when cleavable cells undergo extensive intrinsic and/or extrinsic damage, including oncogenic activation, mitochondrial dysfunction, radiation damage, oxidative and genotoxic stress, and chemotherapeutic agent-induced damage.^8^ At the cellular level, it has been determined that there is a relationship among senescence, CIN and aneuploidy in various cell types in different species.^9^ Interestingly, increasing evidence supports a connection between CD and aging, indicating that centrosomes may have direct or indirect effects on senescence.^10^

According to the results of Boveri’s research a century ago, centrosome numerical abnormalities may result in tumorigenesis.^11^ Boveri created fertilized eggs harboring extra centrosomes, and these eggs appeared to undergo multipolar mitoses. In Boveri’s model, centrosome abnormalities caused chromosome mis-segregation during mitosis, which triggered malignancy. Importantly, increasing amounts of circumstantial evidence have suggested that aberrant centrosome numbers and functions are associated with aneuploidy and human cancers,^3^ and the causal relationships between centrosome abnormalities and tumorigenesis have been characterized.^12,13^ Centrosome abnormalities in cell culture often result in aberrant spindle assembly and damaged microtubule–kinetochore attachments.^14^ As demonstrated by Jusino et al., centrosome anomalies and tumorigenesis are connected in that centrosome dysregulation leads to persistent CIN.^15^ Interestingly, chromosome segregation errors and replication stress resulting from CIN can generate double-stranded DNA (dsDNA) and revitalize the innate immunological response by activating cyclic GMP–AMP synthase (cGAS)–stimulator of interferon genes (STING) signaling.^16,17^ This recent discovery not only enhances our understanding of the effects of CD on tumor development but also elucidates the consequences of CD associated with the interplay between cancer cells and the immune microenvironment. Here, we explore the impacts of such recent advances on our current understanding of the causes and consequences of centrosome abnormalities with regard to senescence and tumor immunity.

## Mechanisms of centrosome abnormalities

**Fig. 1:**
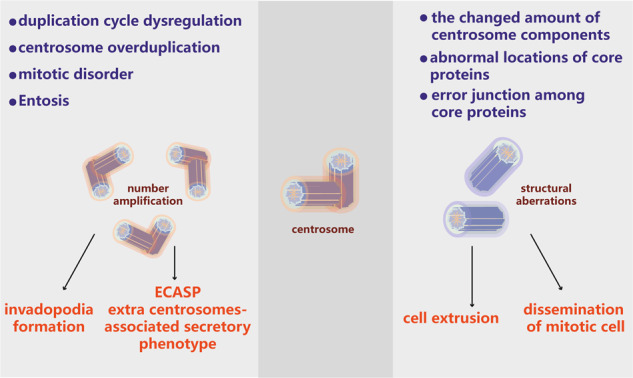
Mechanisms of centrosome aberrations in cancer. The mechanisms of centrosome number abnormalities include duplication cycle dysfunction, centrosome overduplication, mitotic disorder and entosis. These processes result in the formation of invadopodia and establishment of an extra centrosome-associated secretory phenotype (left). The mechanisms of structural centrosome abnormalities include changes in the amounts of centrosome components, abnormal localization of core proteins, and aberrant binding among core proteins; these changes lead to cell extrusion and dissemination of mitotic cells (right)

Increasing amounts of evidence have shown that centrosome abnormalities in cells are fairly universal among solid tumors and in many hematopoietic malignancies. For example, approximately 80% of invasive breast tumors exhibit amplified centrosomes.^18^ Consistent with these findings, centrosome abnormalities have been detected in 72% of patients with B-acute lymphoblastic leukemia.^19^ Importantly, centrosome amplification is strongly associated with the development of aneuploidy and CIN in a variety of tumor types.^18–21^ Centrosome abnormalities in tumors can be roughly divided into numerical abnormalities and structural abnormalities (Fig. 1). Increases in centrosome copy numbers cause numerical abnormalities; such increases can be caused by mistakes in centrosome replication or by failure of cell division. Centrosome numbers have been found to be significantly associated with tumor grade and the proliferative index in myeloid leukemia, non-Hodgkin’s lymphoma and multiple myeloma.^22–24^ Although the characteristics of structural centrosome abnormalities have rarely been addressed, the most common defect is an increase in centrosome size due to PCM expansion.^25^ In malignant breast cancer, structural centrosome abnormalities involve mainly overly long centrioles that enhance centrosome amplification through both centriole fragmentation and ectopic procentriole formation.^26^ In addition, structural centrosome aberrations induced by ninein-like protein (NLP) can induce reorganization of the cytoskeleton and increase cell stiffness to trigger cell dissemination.^27^ Although structural and numerical abnormalities often coexist in tumors and induce some of the same effects, they can also facilitate different cell mechanistic behaviors, as discussed below.^27,28^

## Numerical centrosome abnormalities

Numerical abnormalities, including centrosome amplification, are the most common centrosome defects in tumors. Centrosome amplification can be induced through many mechanisms, such as cytokinesis failure, mitotic slippage, cell–cell fusion, centriole overduplication and de novo centriole assembly.^29^ Various potential mechanisms can explain the variety of proteins, such as tumor suppressors and oncogenic proteins, involved in centrosome amplification in the context of cancer.^30^ Importantly, centriole overduplication, rather than cytokinesis failure or cell–cell fusion, is considered the predominant contributor to centrosome amplification, as indicated by the results of an analysis of centrosomes in primary human melanomas. In that study, CEP170 staining was used to assess whether centrosomes contained mature centrioles. Overduplicated centrioles were expected to not harbor CEP170, while extra centrioles resulting from cell doubling events were expected to harbor CEP170. The authors found a lower percentage of CEP170-positive centrosomes in melanomas than in benign samples.^31^

Dysregulation of the centrosome duplication cycle is one of the crucial pathways leading to centrosome amplification. The duplication cycle is partially, but strictly, regulated by many pivotal regulators; in normal cells, centrosome amplification is prevented by numerous factors that regulate centrosome duplication positively or negatively.^32^ However, this strictly controlled centrosome cycle depends on only a few evolutionarily conserved core proteins.^33^ For example, polo-like kinase (Plk) 4 is an important core protein that is a major regulator of centrosome duplication.^34^ The activity of Plk4 is a key factor in the regulation of centriole number, as indicated by the findings that excessive activity of Plk4 results in additional centrioles,^35^ while Plk4 depletion decreases centriole numbers.^35,36^ To guarantee correct centriole duplication, Plk4 levels are primarily regulated by SCFβTrCP/ubiquitin-dependent proteolysis,^37^ which is partially controlled by autophosphorylation.^38,39^ P53 negatively regulates Plk4 mRNA levels by recruiting histone deacetylase (HDAC) repressors to the promoter of Plk4.^40^ Therefore, loss of p53 may promote centrosome amplification by increasing the levels of Plk4. This mechanism is consistent with the observation that deletion of p53 is related to increased centrosome numbers in mouse fibroblasts.^41^ However, a recent study has demonstrated that the numbers of centrosomes in the brains of p53^–/–^ mice are normal,^42^ showing that deletion of p53 is insufficient to cause centrosome amplification. Therefore, p53 may affect amplification differently in different tissues, suggesting that other mechanisms may be more influential than p53 signaling with regard to centrosome amplification.^43^

Tetraploidy, which can be caused by abnormal centrosome replication or by failure of cell division, was first discovered in fertilized eggs by Theodor Boveri.^11^ Notably, previous studies have revealed that centrosome amplification occurs in cells with telomere-driven tetraploidy, which can result from cytokinesis failure, mitotic slippage, endoreduplication or cell–cell fusion and can promote transformation.^44,45^ Although these studies did not evaluate the effects of centrosome amplification, cells originating from p53^–/–^ tetraploid tumors have been found to have increased numbers of centrosomes.^46^ In one study, tetraploid cells isolated from p53^–/–^ mouse mammary epithelial cells (MMECs) undergoing transient cytokinesis failure caused mammary epithelial tumorigenesis upon subcutaneously implantation into nude mice.^46^ However, some findings of a recent in vitro study contradict the supposition that tetraploid tumors exhibit centrosome overduplication: specifically, tetraploidized p53^–/–^ HCT116 cells fail to cleave and cease to proliferate, resulting in long-term loss of centrosomes in cultured cells.^47^ One possible reason for this result is that transient blockade of cytokinesis may generate aneuploid cells and subsequently lead to propagation of both diploid and tetraploid p53^–/–^ cells. On the other hand, the oncogene Mos plays a pivotal role in multipolar mitoses of p53^–/–^ cells. Mos inhibits the coalescence of supernumerary centrosomes to induce frequent tetraploidization, and knockdown of Mos stagnates multipolar mitoses and drives CIN in p53^–/–^ cells.^48^ Taken together, these findings indicate that there is not a simple correspondence between the production of additional centrosomes and the continued maintenance of extra centrosomes. Clearly, centrosome amplification itself is detrimental. Previous studies have shown that additional centrosomes disappear spontaneously in newly originated tetraploid cells during continuous passage in culture.^49^ In addition, other permissive conditions must coexist, such as conditions related to specific cell types and genetic changes, for cytokinesis failure to induce tetraploidy in cells and thus to cause long-term stable centrosome amplification.

Notably, entosis is observed in cells found in the urine and ascites fluid of cancer patients^50^ and is identified by the presence of cell-in-cell structures.^51^ Entosis occurs mainly in epithelial tumor cells under specific conditions, such as abnormal proliferation, glucose exhaustion,^52^ matrix detachment,^51^ and mitotic stress.^53^ The effects of entosis on tumorigenesis remain unclear; however, entosis may induce aneuploidy as cells are engulfed^54^ and may supply nutrition for tumor growth.^52^ Cell division, detachment or death are the likely ends for engulfed cells. However, deficiencies in autophagosome–lysosome function and apoptosis can cause internalized cells to be released by their host cells and reappear unharmed.^51,55^ In human breast tumors, live cells are internalized by entosis, but the centrosomes of native cells are preserved in the host cells. Furthermore, the preserved centrosomes continue to function during the cell cycle of the host cells, thereby disrupting the formation of the contractile ring during host cell division. Consequently, cytokinesis failure occurs frequently, producing binucleate cells that induce increases in centrosome number.^54^ These observations suggest the existence of a novel relationship between cytokinesis failure and centrosome abnormalities.

## Structural centrosome abnormalities

Centrosome structural defects can be roughly classified into two groups: defects in centriole structure and defects in the amounts of PCM components (Fig. 1). Alterations in centriole size are the most straightforward structural defects and are usually observed as increases in centriole length. How centriole structural defects arise in cancer cells remains unclear; however, a recent study has demonstrated that centrosome amplification can be triggered by severe centriole overelongation, which results in the formation of overactive centrosomes that nucleate more microtubules than normal centrosomes.^26^ Alterations in the expression levels of genes that control centriole structure may underlie structural defects. For instance, upregulation or downregulation of the expression of centrosomal components may result in abnormalities in centriole structure; in a few model systems, overexpression of CPAP/SAS-4 has been found to increase centriole length, which influences normal centromeric assembly.^56,57^

It is not easy to identify specific structural defects of centrioles in cancer cells. First, given that centrioles are 0.2–0.5 μm long, close to the optical resolution limits of optical microscopes, measurement of centriole length requires specific fluorescence techniques or electron microscopy. Second, it is difficult to categorize tumors on the basis of their structural defects. For instance, the amount of PCM could be deduced from the volume/diameter of a centrosome using a pericentriolar marker,^58^ and an increased amount of PCM has been regarded as a structural defect.^59^ However, there are two explanations for increased amounts of PCM in cells. One the one hand, an increase in PCM could be a valid interpretation of the measured data and support the classification of excess PCM as a centrosome structural defect.^58^ On the other hand, the observation could be a result of additional centrosome clustering during interphase, which is considered a numerical defect.^58,60^ These two possibilities can be assessed only by authentic centriole labeling. Incorrect and opposite categorization can also occur because it is thought that increased centriole length results in centriole fragmentation in vitro.^57^ Therefore, determining the origins of centrosome structural changes via only fixed-cell imaging is not an easy task and is further complicated by the fact that most tumor studies consider only PCM markers without considering centriole markers. Most importantly, it is necessary to systematically identify and classify centrosome abnormalities through specific methods in order to better describe centrosome abnormalities in human tumors and elucidate how these centrosomal defects arise.

## Centrosome aberrations and senescence

**Fig. 2:**
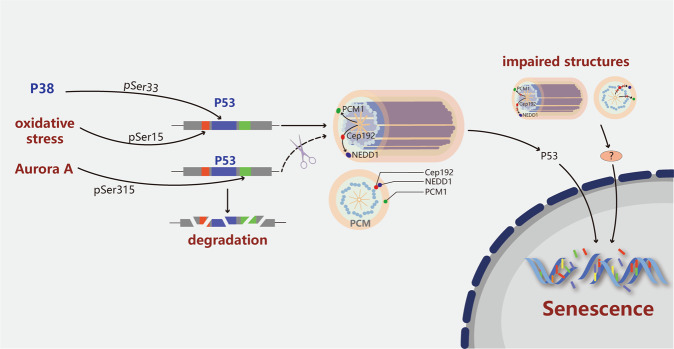
Centrosome aberration-associated molecular pathways in cellular senescence. Oxidative stress and several stress-associated regulators activate p53 via phosphorylation (forming phosphorylated p53, P-p53) to stimulate the translocation of p53 to centrosomes or promote its degradation. Phosphorylation of centrosomal p53 subsequently triggers the activation of proteins that modulate the onset of senescence. In addition, dissociation of pericentrin or PCM-1 induces permanent exit from the cell cycle that is accompanied by an increase in the expression of cellular β-galactosidase, a hallmark of cellular senescence. Similarly, disruption of other PCM components, such as Cep192 (which recruits NEDD1 to the PCM) and NEDD1 (which recruits γ-tubulin to the PCM), leads to centrosome fragmentation and premature entry into the senescence pathway. The scissors indicate that p53 cannot translocate into centrosomes

Senescence, or aging, is a natural biological process that occurs in all living organisms. The characteristics of aging include disruptions to cellular metabolism and function that change with time to ultimately cause permanent cell cycle arrest and cell death. Although senescence is observed both in whole organisms^61,62^ and in individual cells,^63^ the molecular and cellular mechanisms of senescence are still unclear. At the cellular level, it has been determined that senescence is correlated with CIN and aneuploidy in different cell types in different species.^9,64^ Some physiological stresses like oxidative stress are considered to play roles in the aging process,^65^ DNA damage,^66^ telomere shortening,^67^ high levels of tumor suppressor gene expression^68^ and oncogenic activation.^69^ Interestingly, increasing evidence has revealed a relationship between CD and senescence (Fig. 2), suggesting that the centrosome could play a role in aging directly or indirectly. For example, aged porcine oocytes exhibit loss of γ-tubulin and NuMA, which are critical PCM components of the meiotic spindle; this loss results in increased spindle abnormality and disorganization.^70^ In the same way, the spindles of aged human oocytes gradually lose microtubules, which strongly suggests that centrosome structure and function are impaired during aging.^71^ In addition, the integrity of centrosomes and microtubules is lost in aged human oocytes^72^ and in aged Drosophila cells.^73^ Moreover, because of telomere shortening and oxidative stress, human primary fibroblasts no longer divide and instead enter replicative senescence after a limited number of cell divisions.^63^ These cells become senescent due to increases in the frequency of abnormal mitosis and the incidence of supernumerary centrosomes.^9^ Importantly, disruption of core PCM components in early-passage mouse embryonic fibroblasts (MEFs) can induce centrosome fragmentation and initiate premature senescence,^74^ indicating that CD alone is enough to promote the progression of cellular senescence. Another study has revealed that pericentrin and PCM-1 play roles in cell cycle regulation.^75^ PCM-1 can recruit pericentrin to PCM, and inhibition of pericentrin or PCM-1 induces permanent exit from the cell cycle that is accompanied by increased expression of cellular β-galactosidase, a hallmark of cellular senescence. Similarly, depletion of other PCM components, such as Cep192 (which recruits NEDD1 to the PCM) and NEDD1 (which recruits γ-tubulin to the PCM), leads to centrosome fragmentation and premature entry into senescence.^73^ In summary, these findings show the interesting possibility that centrosome aberrations are types of cellular stresses that can prime cells to exit the cell cycle permanently. Future high-resolution and electron microscopy studies are necessary to identify these structural aberrations and determine their contributions to senescence.

The underlying molecular mechanism of CD and its effects on cellular senescence induced by various pathological stresses have recently been explored. As shown in Fig. 2, oxidative stress induces replicative senescence of primary MEFs with age;^65^ however, instead of increasing in number, the centrosomes are fragmented into smaller pieces as late-passage cells begin to undergo senescence.^74^ Moreover, centrosome amplification induces an early oxidative stress response by increasing NOX-mediated generation of reactive oxygen species (ROS), and high ROS levels induce a senescence-like phenotype in cells with additional centrosomes.^28^ P38 is a protein involved in the cellular stress response and senescence; intriguingly, p38 is activated to phosphorylate p53 at Ser33, which causes p53 to accumulate at centrosomes before it is translocated to the nucleus.^76^ Similar to the findings of previous studies, this finding indicates that cell cycle arrest also depends on p38 and p53 and that the arrest may be due to increased protein levels of p53 and p21 and decreased levels of phosphorylated retinoblastoma (Rb). Moreover, Aurora A and its downstream target, TACC3, localize to the PCM during mitosis. K162R mutation inactivates Aurora A, and inhibition of either Aurora A or TACC3 results in premature senescence of p53-proficient tumor cells by increasing p53, p21 and hypophosphorylated Rb levels.^77^ The increased p53 levels may be partially explained by findings that Aurora A typically phosphorylates p53 at Ser315 to make it sensitive to degradation and that p53 becomes stable in the absence of Aurora A.^78^ Primary human fibroblasts undergoing replicative senescence or premature senescence induced by oxidative stress also accumulate p53 in the centrosome and exhibit simultaneous p53 phosphorylation at Ser15.^79^ Phosphorylation of p53 at Ser15 is critical not only for p53 localization to the centrosome but also for the default pathway of early mitosis that ensures proper cell division through ataxia telangiectasia mutated (ATM) at the centrosome.^80^ When the mitotic spindle performs normal function, Ser15 is rapidly dephosphorylated, and p53 remains isolated and inactive at the centrosome. However, when the spindle is impaired, p53 remains phosphorylated at Ser15; eventually, the phosphoprotein is translocated to the nucleus to trigger cell cycle arrest and cell senescence. Thus, it is possible that the phosphorylation and accumulation of p53 at the centrosome is a pivotal event that occurs early in the process of senescence in response to centrosome damage and other stresses. It has long been known that p53 is located on centrosomes; however, the function of p53 on these organelles is poorly understood.^79–81^ It will be interesting to determine the functional significance of centrosomal p53 and its differential phosphorylation states with respect to various kinases in the future. Elucidating these molecular characteristics will aid in understanding of how p53 integrates signals from different types of stresses to facilitate cellular senescence.

## Centrosome amplification and tumor immunity

**Fig. 3:**
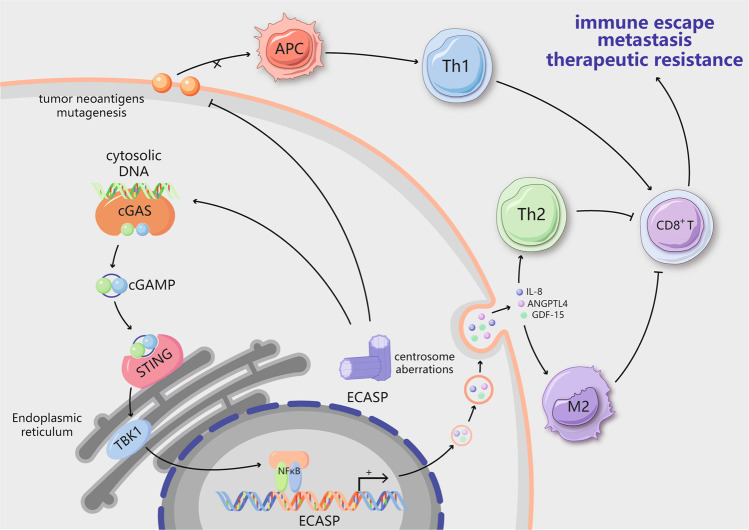
Centrosome aberrations trigger an immunosuppressive microenvironment. Centrosome aberrations can result in the accumulation of double-stranded DNA (dsDNA) in the cytosol. The presence of cytosolic dsDNA activates the cGAS–STING pathway. In cancer cells with centrosome dysfunction, however, alternative inflammatory STING-dependent signaling, such as NF-κB signaling, is activated. Chronic NF-κB activation has been shown to mediate the extra centrosome-associated secretory phenotype (ECASP) by affecting proteins including IL-8, GDF-15 and ANGPTL4. IL-8 is also one of the components of the senescence-associated secretory phenotype (SASP); it recruits Th2 cells and M2 macrophages to shape the immunosuppressive microenvironment. Additionally, centrosome aberrations contribute to decreased tumor neoantigen expression and mutagenesis, thereby suppressing MHC class I antigen presentation and decreasing CD8+ T cell infiltration to enable evasion of antitumor immune responses. Ultimately, centrosome abnormalities can lead to immune escape, distant metastasis and therapeutic resistance

Increasing attention is being paid to the application of effective immunotherapies for the clinical treatment of tumor patients. Molecular identification of tumor antigens is the basis of contemporary tumor immunology and cancer immunotherapy.^82^ A few studies have focused on the impacts of CD on antitumor immune responses and the underlying molecular mechanisms (Fig. 3). Importantly, centrosome status not only regulates aneuploidy development but also controls faithful chromosomal inheritance.^18,20^ Moreover, some observations have indicated that CD can also induce an altered immune phenotype.

With regard to aneuploidy, Senovilla et al. first discovered the connection between nonphysiological tetraploidy and immunosurveillance. In immunodeficient mice, elevated exposure of calreticulin (CALR) on the cell membrane endows hyperploid cancer cells with immunogenicity, possibly by constitutively increasing endoplasmic reticulum (ER) stress. CALR facilitates dendritic cell recognition of neoplasm antigens, which ultimately induces an antitumor immune response and results in suppression of tumor growth via cytotoxic CD8+ T cells. In contrast, hyperploid cells can generate tumors only after a delay in immunocompetent mice; the resulting tumors exhibit reduced DNA content, ER stress, and the exposure of CALR.^83^ Hence, aneuploidy enables cells to escape from the immunosurveillance system. Careful analysis of the components involved in the immune microenvironment has revealed that aneuploidy is positively associated with the overall tumor leukocyte fraction and is consistent with the activation of inflammatory signaling pathways. Macrophages are the most important components of the aneuploid tumor microenvironment, which is distinguished by an immunosuppressive phenotype that involves activation of tumor growth factor-β (TGF-β).^84^ Moreover, highly aneuploid tumors decrease CD8^+^ T cell infiltration to reduce the response to immunotherapy.^85^ Likewise, tumors with aneuploidy exhibit suppressive MHC class I antigen presentation and defective immunogenicity to evade antitumor immune responses.^86^ These interesting correlations require to establish animal models that can be used to dissect the reciprocal crosstalk between aneuploid tumor cells and the corresponding immune microenvironment.

The effects of centrosome amplification on CIN have been elucidated. In tumor tissues, centrosome abnormalities in both size and number are significantly positively correlated with CIN independent of p53 mutation.^18,87^ A study conducted to define the underlying mechanism indicated that extra centrosomes tend to promote chromosome mis-segregation during bipolar cell division.^49^ Subsequently, many research groups have described a direct mechanism through which errors related to mitotic CIN generate DNA breaks via the formation of structures called micronuclei during anaphase arrest.^88^ The envelopes surrounding these micronuclei are prone to rupture, which causes the genomic contents to be exposed to the cytosol.^89^ dsDNA in micronuclei activates the cGAS–STING pathway when dsDNA makes contact with the cytosol during interphase.^16^ cGAS first detects cytosolic dsDNA, causing cGAMP (a cyclic dinucleotide) to be generated, which in turn promotes STING perinuclear localization to the ER membrane^90^ (Fig. 3). STING mediates the transcriptional activation of inflammatory pathways, including the type I interferon signaling pathway, and establishment of the senescence-associated secretory phenotype (SASP). Through fluorescence-activated cell sorting (FACS) and subsequent single-cell RNA sequencing of RNaseH2^–/–^ MEFs, Mackenzie et al. elegantly revealed that proinflammatory interferon-stimulated genes (ISGs), including CCL5 and CXCL10, are induced only in cells with micronuclei.^91^ Moreover, chromosome-tracking experiments have shown that the same chromosomes undergoing aberrant separation are eventually fragmented into cytoplasmic chromatin,^16^ indicating a direct connection between chromosome mis-segregation and innate immune signaling. Taken together, these findings indicate that CIN caused by the centrosome could activate immune signaling pathways via the cGAS–STING pathway.

## The ECASP links senescence with the immune microenvironment

Recently, the extra centrosome-associated secretory pathway (ECASP) has been identified as a distinct secretory phenotype including diverse extracellular matrix (ECM)-associated factors generated by extra centrosomes.^28^ It has also been demonstrated that centrosome amplification facilitates the release of proinvasive factors to trigger non-cell-autonomous invasion^28^ (Fig. 3). Conditioned media derived from cells with multiple centrosomes can promote the generation of invadopodia in normal cells. Furthermore, cells with additional centrosomes have been shown to release a variety of proinvasive factors that are associated with tumorigenesis and with the invasion and migration of cancer cells (e.g., IL-8, ANGPTL4, and GDF-15). The release of proinvasive factors does not require Rac1 signaling; however, Rac1 signaling is required for the formation of invadopodia in response to these secreted factors in cells with normal centrosomes. This finding indicates that the non-cell-autonomous ECASP differs from the previously reported pathway that promotes the cell-autonomous formation of invadopodia in cells with additional centrosomes.^60^ Although how the additional centrosomes promote the ECASP remains unknown, it has been determined that the response depends in part on increases in the levels of ROS in centrosome-amplified cells.^28^ Similarly, aging cells generate a complex mixture of soluble and insoluble factors that comprise the SASP,^92^ including cytokines, chemokines and other signaling molecules. The specific constitution of the SASP varies depending on the type of cell and the agent inducing senescence. IL-8 is one of the components of the SASP and can promote invasion of the basement membrane by precancerous epithelial cells.^93^ This suggests that IL-8 may connect the ECASP with the SASP. The mechanism involves activation of cGAS/STING and NF-κB and accumulation of these proteins in chromatin components, which may be the point of convergence for centrosome abnormalities, the immune microenvironment and senescence. Accumulating evidence has indicated that tumors can achieve tolerance via various mechanisms, including through adapting their response to cytosolic DNA signaling or obtaining genomic copy number heterogeneity, to avoid activating harmful components of innate immunity. Although tumor cells may inhibit type I interferon signaling induced by cytosolic DNA, activation of STING facilitates other inflammatory pathways in a tumor cell-autonomous manner. Despite the lack of a significant association between STING and ISGs, there is a significant association between the mRNA levels of cGAS and STING and a positive association between the expression of these genes and the SASP,^94^ which is consistent with the reported effect of the cGAS–STING pathway on cellular senescence.^95^ STING facilitates signaling through many proinflammatory transcription factor-related pathways, such as the canonical and noncanonical NF-κB pathways.^96^ Although the functions of these factors overlap, different outcomes can result from their various effects downstream of STING. For example, the classic NF-κB pathway triggers the generation of SASP inflammatory factors through cytosolic DNA signaling in primary human lung IMR90 fibroblasts.^94,95^ Additionally, through low-level and persistent activation of cGAS–STING, the noncanonical NF-κB pathway is enhanced and has a crucial effect on the migration and invasion ability of MDA-MB-231 cells.^16^ These pathway-specific functions do not seem to be associated with interferon-regulatory factor 3 (IRF3). Although the role of the ECASP in tumor development remains unknown, this pathway participates in a mechanism by which additional centrosomes trigger the paracrine invasion of nearby cells with normal centrosomes.^28^ A small fraction of tumor cells undergoing chromosome mis-segregation may be required to generate SASP-associated cytokines that attract immune cells to the tumor microenvironment. The inflammatory microenvironment could in turn result in aneuploidy and propagate CIN in tumor cells via direct genotoxic stress or induction of epithelial–mesenchymal transition (EMT), forming a feed-forward loop.^97^ It would be interesting to explore the consequences of structural centrosome alterations in order to determine whether they are similar to those of numerical alterations that induce a pro-invasive secretory phenotype.

## Centrosome abnormalities and cancer therapy

The recent development of centrosome clustering inhibitors might enable validation of the role of centrosomes in cancer in vivo. Some compounds that trigger multipolar mitosis preferentially in cancer cells, such as GF-15 (a derivative of griseofulvin), poly(ADP-ribose) polymerase (PARP) inhibitors and paclitaxel (i.e., Taxol), have been developed^98–111^ (Table 1). Cells with additional centrosomes exhibit increased sensitivity to such inhibitors, but it is uncertain whether the amplification of centrosomes in tumor cells causes this sensitivity. Another possibility is that these agents impact microtubule dynamics to lead to the development of multipolar spindles rather than causing the production of extra centrioles. Taxanes, natural antitumor drugs that have been shown to stabilize microtubules, obstruct cell cycle progression by inducing centrosome-related abnormalities, specifically aberrant spindles, and by inhibiting spindle microtubule dynamics.^110^ Paclitaxel, a prototypical taxane antitumor drug that inhibits tubulin polymerization, can induce abnormal multipolar spindle formation to sustain cleavage failure and cause gradual cell death.^109^ Griseofulvin has many properties in common with paclitaxel. Griseofulvin facilitates microtubule instability at low concentrations, and the griseofulvin binding site on tubulin is the same as the Taxol binding site, thereby indicating that griseofulvin has similar effects in inducing multipolar spindle formation.^108^ GF-15, a derivative of griseofulvin, potently inhibits centrosomal clustering, thereby suppressing tumor cell growth in vitro and in vivo.^100^ In addition, a quinoline–sulfonyl hybrid proteasome inhibitor, VR23, targets the catalytic β2 subunit of the 20S proteasome and induces an aberrant centrosome amplification cycle by promoting the accumulation of ubiquitinated cyclin E to selectively kill cancer cells.^107^ Furthermore, centrosome clustering chemical inhibitor-01 (CCCI-01) induces multipolar spindle formation and inhibits clonogenic growth of BT-549 breast cancer cells, which have extra centrosomes but retain bipolar spindles similar to those in normal epithelial cells.^106^ Likewise, CP-673451 and crenolanib have been discovered to be robust inhibitors of centrosome clustering with selective cytotoxicity toward cells with extra centrosomes. Mechanistically, both compounds induce mitotic spindle multipolarity via cofilin-mediated cortical actin destabilization.^105^ Given the key roles of PLKs in centrosome maturation, some targeted PLK1 and PLK4 inhibitors have been developed to significantly inhibit tumor growth and are currently in the clinical development stage.^103,104^ Ultimately, PARP-1 localizes to centrosomes and catalyzes poly(ADP-ribosyl)ation of centrosomal p53, which is involved in regulation of centrosome duplication and monitoring of chromosomal stability.^112^ Moreover, PARP inhibitors have been discovered to inhibit centrosome clustering and exclusively eradicate multicentrosomal human cancer cells.^98,111^ Hence, PARP inhibitor treatment could be a new, selective and efficient centrosome-targeting therapy for a wide range of human cancers.

**Table 1:** Potential centrosome-targeted therapy in cancer

Agent	Mechanisms	Preclinical and clinical effects	Reference
Taxanes paclitaxel	Inhibits tubulin polymerization and indirectly induce multipolarity spindles	A chemotherapy medication used to treat a number of types of cancer	Abal et al.^110^ Zhu et al.^109^
GF-15	Inhibits centrosomal clustering	Inhibits the proliferation of tumor cells in vitro	Raab et al.^100^ Zacharaki et al.^108^
VR23	Targets the catalytic β2 subunit of the 20 S proteasome and induces an aberrant centrosome amplification cycle	Kills multiple myeloma cells and metastatic breast cancer cells in vitro and in vivo, and enhances the antitumor activity of paclitaxel	Pundir et al.^107^
CCCI-01	Blocks centrosome clustering	Recedes the tumor growth in vitro	Kawamura et al.^106^
CP-673451 crenolanib	Cofilin-mediated cortical actin destabilization	Inhibit the tumor growth in vitro	Konotop et al.^105^
CFI-400945	A potent and selective PLK4 inhibitor	Significantly inhibits tumor growth in vitro and in vivo	Mason et al.^104^
SK461364A TKM-080301 GW843682 purpurogallin poloxin	PLK 1 inhibitor	Significantly inhibits multiple tumor growth in vitro and in vivo are being evaluated in phase I or II study	Liu et al.^103^
Olaparib Phenanthrene AZ0108	PARP inhibitors block centrosome clustering	Improve progression-free survival in women with ovarian cancer; enhances the therapeutic efficacy of immune checkpoint blockade	Stewart et al.^102^ Shen et al.^101^ Castiel et al.^98^ Johannes et al.^111^

Aside from acting through these well-established mechanisms of cellular action, inhibitors can affect chromosome segregation by enhancing cGAS–STING pathway signaling, resulting in antitumor immunity. Zierhut et al. showed that STING is an essential determining factor of mitotic cell death in Taxol-treated breast cancer cells in vitro.^113^ Similarly, recent studies have revealed that PARP inhibitors can affect cGAS–STING signaling and antitumor immunity, as indicated by assessments of the tumor response in mouse models of transplantable ovarian and colorectal cancers.^101,102^ Regardless of the associated challenges, it is obvious that identification of agents that rapidly and precisely target centrosomes will be clinically beneficial for certain patients. Additionally, paclitaxel and PARP inhibitors have been shown to enhance immune checkpoint blockade in multiple cancers.^114,115^ Hence, targeted centrosome treatments can promote antitumor responses, and combination therapies are a promising new avenue.

## Conclusion and perspective

The effect of centrosomes on senescence is an important research topic that has been widely ignored. Although further studies are needed, the existing evidence suggests that CD is associated with cellular senescence. We suggest that CD is another form of stress, in addition to well-known cellular stresses such as DNA damage, oxidative stress, oncogenic activation and tumor suppressor overexpression, that can prevent cell cycle arrest and cellular senescence. Notably, increasing amounts of evidence have indicated that p53 is a common protein among these diverse pathways. In response to different sources of stress, p53 accumulates rapidly in the centrosome. Subsequently, phosphorylation of centrosomal p53 by different kinases at specific residues mediates downstream events, including p21 and Rb stimulation, to cause permanent cell cycle arrest and cellular senescence. Although the regulatory role of nuclear p53 in the transcription process is well understood, the biological function of this protein in the centrosome is worth further study. For example, the mechanisms by which p53 shuttles into and out of the centrosome and the specific effect of p53 on senescence deserve further investigation. In addition, whether activation of the autophagic response can delay CD-mediated senescence warrants investigation. Exploration of these topics will likely provide useful insights into the relationships among cellular senescence, cell death, and uncontrolled cell growth, which are all closely associated with proper centrosome function.

Understanding the dual role of CD as both an innate immune signaling activator and a tumor adaptation mediator is crucial for the development of appropriate therapeutic targets. Although recent findings have improved our understanding of the mechanisms underlying CD in biological systems, the clinical application of CD-targeted therapies is only in the beginning stages. Further advances in CD research are essential for enhancing our ability to prevent tumor cells from adapting to cytosolic DNA and to provide therapeutic benefits by targeting the lethal features of cancer. Furthermore, successful treatment hinges on the development of drugs that can activate autophagy to remove abnormal centrosomes and promote antitumor immune responses. Given the widespread distribution of CD in human cancers, CD-targeted therapies likely have the capacity to improve clinical outcomes, such as by minimizing therapeutic resistance, ameliorating advanced and metastatic disease and enhancing systemic antitumor immunity.
